# The Performance of Olfactory Receptor Neurons: The Rate of Concentration Change Indicates Functional Specializations in the Cockroach Peripheral Olfactory System

**DOI:** 10.3389/fphys.2020.599086

**Published:** 2020-12-23

**Authors:** Harald Tichy, Marlene Linhart, Alexander Martzok, Maria Hellwig

**Affiliations:** Department of Neurosciences and Developmental Biology, Faculty of Life Sciences, University of Vienna, Vienna, Austria

**Keywords:** food odor coding, differential sensitivity, resolving power, electrophysiology, cockroach

## Abstract

Slow and continuous changes in odor concentration were used as a possible easy method for measuring the effect of the instantaneous concentration and the rate of concentration change on the activity of the olfactory receptor neurons (ORNs) of basiconic sensilla on the cockroach antennae. During oscillating concentration changes, impulse frequency increased with rising instantaneous concentration and this increase was stronger the faster concentration rose through the higher concentration values. The effect of the concentration rate on the ORNs responses to the instantaneous concentration was invariant to the duration of the oscillation period: shallow concentration waves provided by long periods elicited the same response to the instantaneous concentration as steep concentration waves at brief periods. Thus, the double dependence remained unchanged when the range of concentration rates varied. This distinguishes the ORNs of basiconic sensilla from those of trichoid sensilla (Tichy and Hellwig, 2018) which adjust their gain of response according to the duration of the oscillating period. The precision of the ORNs to discriminate increments of slowly rising odor concentration was studied by applying gradual ramp-like concentration changes at different rates. While the ORNs of the trichoid sensilla perform better the slower the concentration rate, those of the basiconic sensilla show no preference for a specific rate of concentration increase. This suggests that the two types of sensilla have different functions. The ORNs of the trichoid sensilla may predominately analyze temporal features of the odor signal and the ORNs of the basiconic sensilla may be involved in extracting information on the identity of the odor source instead of mediating the spatial-temporal concentration pattern in an odor plume.

## Introduction

Any change in odor concentration occurs at a certain rate, and any rate of change takes place between two different concentration values. An insect tracking an odor plume will encounter from one moment to the next not only a succession of different odor concentrations but also different rates of change. When the rate of concentration increase is doubled by keeping the concentration difference and halving the time passed, is the response of the olfactory receptor neurons (ORNs) also twice as strong? Does an increase in the rate of concentration increase determine the ORNs performance in detecting concentration differences along a gradient? There is no definite answer, but electrophysiological experiments with a specific type of ON and OFF ORNs located in morphologically identifiable single-walled trichoid (*swC*) sensilla on the cockroach’s antenna revealed that the differential sensitivity or the gain of responses for the rate of concentration change decreases rather than increases with rising rate of change (Burgstaller and Tichy, 2012; Tichy et al., 2016; Tichy and Hellwig, 2018). The high gain for slow rates of change improves the cockroach’s ability to detect slowly fluctuating or creeping concentration changes which persist in one direction.

Gain control which adjusts sensitivity according to the rate of concentration change has been studied in detail only in the ON and OFF ORNs. Furthermore, pairs of ON and OFF ORNs have been identified so far only in the trichoid sensilla of the cockroach. Could it be that gain control is restricted to the ON and OFF ORNs and the trichoid sensilla, or that it is widely used among ORNs of other types of the cockroach’s sensilla but with different scaling? Here we describe the result of experiments similar to those performed on ORNs of trichoid sensilla, but this time focusing on basiconic sensilla. To facilitate comparison of the data obtained in the two types of olfactory sensilla, we used identical methods of stimulation and evaluation. The specific differences between the gain of responses and the performance in discriminating concentration increments can thus be assigned to properties of the ORNs. We used the odor of lemon oil as stimulus because it contains many different compounds which elicit, when tested separately or as a mixture, strong excitatory responses in several types of ORNs located in basiconic sensilla (Sass, 1978) and also in antennal lobe neurons (Boeckh, 1974; Selzer, 1984; Zeiner and Tichy, 2000). Lemon-odor responsive ORNs were studied in the 1970s and 1980s with electrophysiological techniques, and due to the state of art at that time, odor pulses were used to describe the combinatorial activity patterns underlying encoding of odor identity. One main focus of our present work was to determine whether the ORNs of basiconic sensilla, which in previous reports have been assigned a role in coding the identity of food odors (Sass, 1972, 1976, 1978; Schaller, 1978; Boeckh and Ernst, 1987), also adjust their response gain according to the fluctuating concentration changes of the signal. A further focal point was to provide information on the ability of these ORNs to discriminate concentration increments. Two kinds of experiments are described. In the first, concentration was changed in an oscillating fashion and the duration of the oscillation period was varied, and in the second, concentration was changed in a linear, ramp-like fashion and the steepness of the slope was varied. Oscillating concentration changes provide perfect stimulus conditions to study gain control of the responses to the instantaneous concentration and its rate of change (Burgstaller and Tichy, 2012), whereas ramp-like concentration changes are optimal to investigate the power of resolving increments of continuously rising and falling odor concentrations (Tichy et al., 2016).

## Materials and Methods

### Preparation and Recording

The antenna of a male adult cockroach (*Periplaneta americana*) was strapped onto a Plexiglas holder with Parafilm. For the extracellular recordings from single sensilla, one antenna was attached with adhesive tape and dental cement onto a ledge that extended from the holder. The electrodes were electrolitically sharpened tungsten needles. The reference electrode was inserted in longitudinal direction into the tip of the antenna, and the recording electrode into the base of the sensillum. Action potentials were amplified (NPI, SEC-05X) and filtered (0.1–3 kHz), passed through a micro 1401mkII A-D converter (CED, Cambridge Electronic Design, 12 bit, 167 kHz), and fed into a PC. The digitized action potentials were displayed with the voltage outputs of the electronic flow meters and the miniPID on-line on a monitor, stored on a hard disk, and analyzed off-line using Spike2 software (Version 6, Cambridge Electronic Design).

### Stimulation

Lemon oil is a very effective odor in eliciting activity from antennal ORNs and antennal lobe neurons (Boeckh, 1974; Sass, 1978; Selzer, 1981, 1984). It contains different compounds of several chemical classes (Günther, 1968; Shaw, 1979). The sensory consistency of natural fruits can differ greatly depending upon the regional diversity, ripeness stage, and storage. Therefore, synthetic lemon oil (relative density = 0.85, Art. 5213.1; Carl Roth GmbH + Co. KG; Karlsruhe, Germany) was used as a standardized fruit odor stimulus.

The air dilution flow olfactometer used to apply the odor stimulus was described recently (Tichy et al., 2020). Here we will provide a short overview of the stimulation technique. Clean compressed air was divided into two streams with equal flow rates. One stream flowed through a tank with the undiluted lemon oil. The other stream was led through an empty tank of the same design and remained clean. Then the two air streams passed through electrical proportional valves and electronic flow meters. The two streams were then combined. A 180*°* degree phase shift of the valves’ control voltages (digital analog outputs of micro1401mkII) ensured that the total flow rate of the combined air stream was held constant at 1.5 ms^–1^ as the flow rate ratio of the odor-saturated to clean air varied. This ratio was regulated by means of the output sequencer function of the data acquisition software (Spike2, v.6.), using a self-written sequencer script. The mixed air stream emerged from a nozzle 7-mm in diameter at a distance of 10 mm from the recording site. A suction tube continually removed the air around the antenna. The digitized output voltage of the electronic flow meters, calibrated by the manufacturer for flow rate, was used to monitor the flow profiles of the two individual air streams and of the mixed air stream representing the odor delivery during stimulation. The concentration of the stimulus was determined by the flow rate ratio of odor-saturated air to clean air and expressed as percentages of the total flow rate: “0%” means clean air only and indicates that the air stream directed onto the cockroach does not contain the odor of lemon oil; “50%” means odorized and clean air streams are mixed in a 50:50 ratio. A photoionization detector (200A miniPID, Aurora Scientific) was used to verify the time course of slow concentration changes.

### Response Evaluation

Impulse frequency (imp/s) is the impulse count per second for fixed periods of time (bin widths). To directly compare the ORN responses of basiconic sensilla with those obtained in previous studies for trichoid sensilla, the same bin widths was used: 0.2 s for evaluating the responses to oscillating concentration changes (Burgstaller and Tichy, 2012) and 0.5 s for 5%/s ramps, 0.2 s for 20%/s ramps, and 0.1 s for 50%/s ramps (Tichy et al., 2016).

The differential sensitivity or gain of response is the mean change in impulse frequency per unit change in the instantaneous concentration and the rate of concentration change. This quantity was estimated by the slopes of regression planes approximating the stimulus-response relationship for oscillating concentration changes or parabolic regressions for ramp-like concentration changes. In parabolic regressions, the differential sensitivity is the mean of the first differentials of a given curve at the abscissa values used to stimulate the ORN. The resolving power of an ORN can be determined from the differential sensitivity and the scatter of individual responses. Resolving power is the number of discrete concentration steps that the impulse frequency can distinguish within the tested concentration range. Here it is defined as the difference between two concentrations that a single ORN of average differential sensitivity needs in order to identify the higher concentration with a given high probability (e.g., 90%). A full mathematical development of the concepts underlying the resolving power was presented by Burgstaller and Tichy (2011) and Tichy et al. (2016). The equation is

$$\Delta x=\frac{\sqrt{2\sigma }}{\left|b\right|}\Phi^{-1}\left(y\right)$$

in which |*b*| is the mean absolute slope of the stimulus-response functions, σ^2^ is the variance of individual deviations of points about their respective regression, *y* is the required probability (90%), and Φ^–1^(*y*) is the inverse of the distribution function of a standardized, normally distributed, random variable. Φ^–1^(0.9) = 1.28 (Diem and Lentner, 1970, Tables p. 28). In case of a linear regression,

σis2estimatedbyσ2=∑ε2n-2I,andforaparabolabyσ2=∑ε2n-3I,

where ε is the deviation of each individual point from its curve, *I* is the number of curves, and *n* the number of measurements. *n* is reduced by the number of degrees of freedom, which is *2I* because 2 estimates are necessary to determine each straight line (*a* and *b; y* = *a* + *bx*). Since the resolving power is calculated from parabolas, *n* is reduced by *3I*, corresponding to the 3 estimators for each parabola (*a, b*, and *c; y* = *a* + *bx* + *cx*^2^).

This method can be applied if the following conditions are met: (*i*) the deviations of the individual points from their curves must be normally distributed about a mean of zero, and (*ii*) the absolute deviations (sign ignored) must not depend on the slope of the curves. The absolute deviations of single points from their regressions did not depend on the regression slopes. However, their distribution was not always normal (*x*^2^-test). Though bell-shaped, the flanks of the distribution curve were too steep; the points tended to be located too centrally. This type of distribution will, if anything, underestimate the resolving power. The normal distribution model was accepted for the lack of a better one.

## Results

**FIGURE 1:**
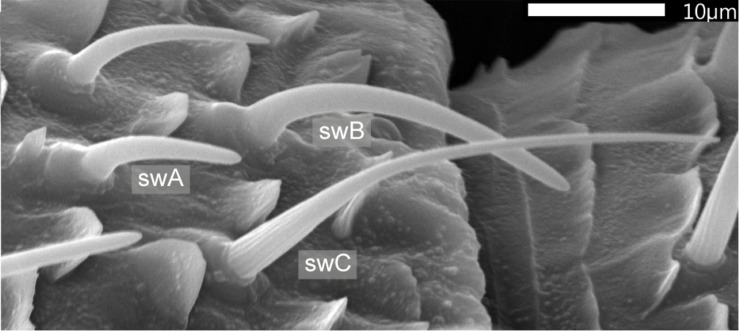
Scanning electron micrograph of the distal edge of a round-shaped segment from the middle antennal region of the male cockroach. Two main types of olfactory sensilla were distinguished according to external features: sensilla basiconica and trichoidea. Based on their wall structures, Schaller (1978) subdivided the basiconic sensilla into single-walled type *A* (*swA*; length, 8–12 μm; base diameter, 2–3 μm) and single-walled type *B* (*swB;* length, 18–28 μm; base diameter, 3–4 μm). The trichoid sensilla include the single-walled type *C* (*swC*; length, 30–40 μm; base diameter, 3 μm) which contains the ON and OFF ORNs. Data taken from Schaller (1978).

Electrophysiological recordings were obtained from two types of single-walled (*sw*) basiconic sensilla which were classified by Schaller (1978) according to size and morphology as short *swA* (length, 8–12 μm) and long *swB* sensilla (18–28 μm) (Figure 1). Both sensilla types house ORNs which respond to the odor of lemon oil presented as brief pulses of high concentration separated by intervals of clean air (Sass, 1978). We performed two kinds of experiments involving slowly oscillating and ramp-like changes of the lemon-oil odor over a range of concentrations between 0 and 50%. Oscillating concentration changes imitate fluctuations in odor concentration, concentration ramps gradually rising concentrations. During oscillating concentration changes the same rate of change occurs at different instantaneous concentrations when the oscillation period varies. It will therefore be possible to determine to what extent each parameter of the odor signal affects the discharge rate. During concentration ramps, on the other hand, the rate of change remains constant when passing different instantaneous concentration values. By varying ramp steepness it will be possible to examine whether or to what extent the resolving power for concentration increments depends on the rate of concentration increase.

In total, 210 basiconic sensilla were examined. Forty-four *swA* sensilla and 40 *swB* sensilla were studied extensively; 10 *swA* sensilla and 7 *swB* sensilla provided the results. We accepted only those ORNs for evaluation that could be readily identified in the recordings and displayed undiminished sensitivity during the experiments.

### Slowly Oscillating Concentration Changes

**FIGURE 2:**
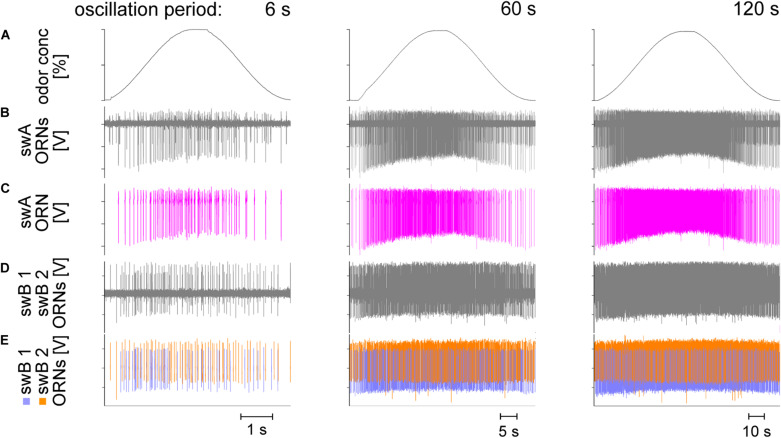
Examples of recordings from a *swA* and *swB* sensillum type during slowly oscillating changes in the concentration of lemon oil odor. Oscillation periods are 6, 60, and 120 s. **(A)** Time course of odor concentration. **(B)** Recordings from the *swA* sensillum showed activities of two ORNs. **(C)** Only one ORN in the *swA* sensillum, producing larger amplitude spikes (*pink*), responded to oscillations in odor concentration. The rate of discharge of the ORN with the smaller impulse amplitudes remained unchanged (not shown in detail). **(D)** Recordings from the *swB* sensillum likewise revealed activities of two ORNs. Unlike the *swA* sensillum, both ORNs were activated by oscillations in odor concentration. **(E)** The ORN responding with the more negative amplitudes (*blue*) was referred to as *swB1* ORN and that with the more positive amplitudes (*orange*) as *swB2* ORN. **(C,E)** Off-line sorted action potentials obtained by spike detecting and template matching techniques using Spike2 software. Note that the increasing density of the action potentials with increasing duration of the oscillation period is due to the decreased time scales in the diagrams.

Figure 2A illustrates three oscillating cycles with periods of 6, 60, and 120 s used to examine the ORNs’ discharge characteristics under slow and continuous concentration changes. The sequence of the concentration oscillations was continuous; each period of concentration change was tested at least three times and the change from one period to the next was continuous. The extracellular recordings from the *swA* sensillum (Figures 2B,C) and the *swB* sensillum (Figures 2D,E) revealed the activity of sets of two ORNs which could be readily distinguished by the shape and amplitude of their impulses. In the *swA* sensillum, only one ORN responded to oscillating concentration changes, in the *swB* sensillum both ORNs. The continuously increasing and decreasing discharge rate of the *swA*-ORNs provides a temporally reliable reproduction of the duration of the oscillation period (Figure 2C). In comparison, the time course of the *swB* ORNs was less orderly and the duration of the oscillation period was less precisely reflected in the discharge rate, particularly during the 6-s period (Figure 2E). Note that during the 120-s period the increase in concentration from 0 to 50% required 60 s at an average rate of 0.8%/s. Thus, the high sensitivity of the ORNs may be an adaptation to slow and continuous concentration changes.

**FIGURE 3:**
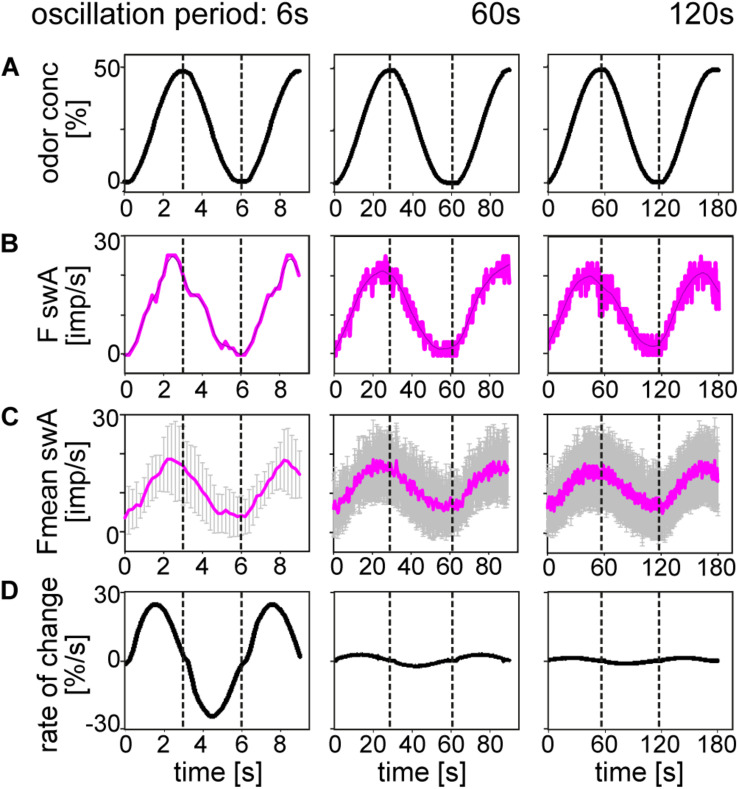
Responses of the *swA* ORN to oscillating changes in odor concentration. **(A)** Time course of odor concentration for three different oscillation periods. **(B)** Time course of impulse frequency. Thin lines are calculated frequency curves smoothing out sharp changes in direction without shifting the maxima and minima values. **(C)** Time course of mean impulse frequency and standard deviations for 10 *swA* ORNs. **(D)** Time course of the rate of concentration change for the three oscillation periods. Different time scales were used to demonstrate the whole oscillation periods. Dotted vertical lines indicate the phase shift between time courses of odor concentration, impulse frequency and rate of concentration change.

**FIGURE 4:**
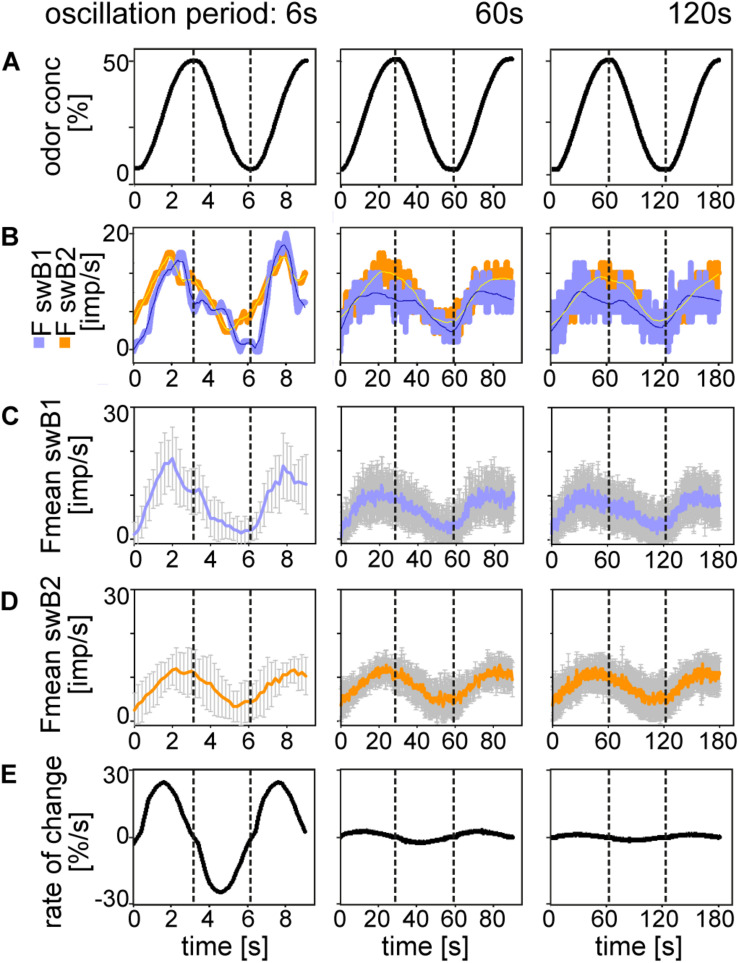
Responses of the *swB1* and *swB2* ORNs to oscillating changes in odor concentration. **(A)** Time course of odor concentration for three different oscillation periods. **(B)** Time course of impulse frequency of the *swB1* ORN (blue) and the *swB2* ORN (orange). Thin lines are calculated frequency curves smoothing out sharp changes in direction without shifting the maxima and minima values**. (C)** Time course of mean impulse frequency and standard deviations for 6 *swB1* ORNs. **(D)** Time course of mean impulse frequency and standard deviations for 7 *swB2* ORNs. **(E)** Time course of the rate of concentration change for the three different oscillation periods. Different time scales were used to demonstrate whole oscillation periods. Dotted vertical lines indicate the phase shift between time courses of odor concentration, impulse frequency and rate of concentration change.

In order to clarify whether there is a correspondence between the ORN discharge rate and the oscillating concentration, we plotted odor concentration and the discharge rates as a function of time (Figures 3, 4). The *top trace* in each diagram represents the time course of the concentration oscillating with periods of 6, 60, and 120 s, and the *second trace* the time course of the corresponding oscillations in impulse frequency. In general, impulse frequency tended to be higher at higher instantaneous concentration values and lower at the lower values. However, the oscillations in impulse frequency and the oscillations in instantaneous concentration were not in step. There was a phase shift between the two, with the frequency curves tending to lead the concentration curves. This phase advance is also apparent in the mean response curves (Figures 3C, 4C,D). The *bottom trace* in each diagram shows the time course of the rate at which concentration changes (Figures 3D, 4E). As the first derivative of instantaneous concentrations, the rate of change was in advance of the oscillating instantaneous concentration. In both shape and position, the frequency oscillations were intermediary, i.e., between those of the instantaneous concentration and the rate of change. This applies to each oscillation period, even when the range of rates of change was smaller. The amplitude of the oscillating frequency of the *swA* ORN (Figures 3B,C) and the two *swB* ORNs (Figures 4B,C) decreased with increasing oscillation period. Nevertheless, the ORNs of both sensillum types displayed a double dependence on the instantaneous concentration and its rate of change. Note that the double dependence of the ORNs’ oscillating frequency reflects the fact that two stimulus parameters are oscillating at the same 1:1 ratio, but their phases are shifted horizontally on the time axis.

**FIGURE 5:**
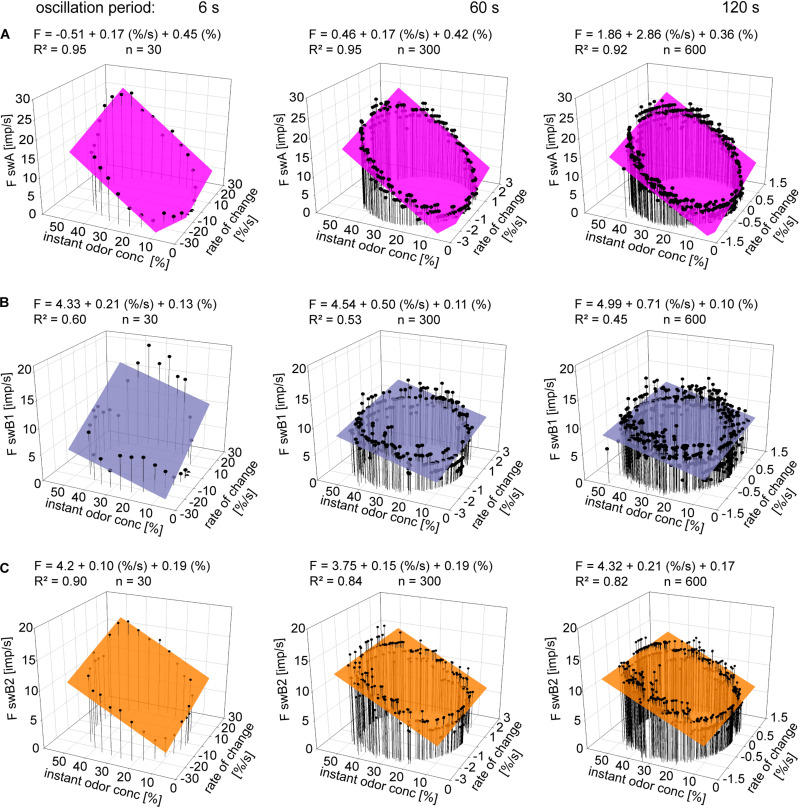
Gain of response for instantaneous concentration and the rate of concentration change. **(A–C)** Impulse frequencies of a single *swA*, *swB1*, and *swB2* ORN during three different oscillation periods of odor concentration plotted as a function of instantaneous odor concentration and its rate of change. Multiple regressions which utilize three-dimensional planes [*F = y0 + a dC/dt) + bC]*; where *F* is the impulse frequency and *y0* is the height of the regression plane] were calculated to determine the gain of the responses for instantaneous odor concentration (*b*-slope) and the rate of concentration change (*a*-slope). *R*^2^, coefficient of determination; *n*, number of points per plot.

To describe the double dependence of the ORNs on the instantaneous concentration and its rate of change, we plotted impulse frequencies of the *swA* ORN and the two *swB* ORNs in Figure 5 as function of the two parameters. The frequency curves display closed, but not perfect, circles similar to Lissajous figures in which two oscillating quantities are plotted one as a function of the other. The shape of the figures produced is determined by the ratio of the frequencies of the two oscillations, the ratio of their amplitudes and their phase differences. Multiple regressions (*F = y0 + a dC/dt + b C;* where *F* is the impulse frequency and *y0* the intercept of the regression plane with the *F* axis, reflecting the height of the regression plane) were used to determine the simultaneous effect of instantaneous concentration (*b* slope) and the rate of change (*c* slope) on the response frequency during different oscillation periods (Figure 5). In the *swA* ORNs, the coefficients of determination (*R*^2^> 0.9) reveal a strong linear relationship between impulse frequency, instantaneous concentration and the rate of concentration change (Figure 5A). In the *swB1* ORNs, the linear relationship (*R*^2^ > 0.8) is moderately strong (Figure 5B), but weak in the *swB2* ORNs (Figure 5C) for the 6 s period (*R*^2^ > 0.6) and non-existent for the 60 and 120 s periods (*R*^2^ > 0.4).

Table 1 provides the means and standard deviations of the gain values or the differential sensitivity of the ORNs. In the *swA* and the two *swB* ORNs, the oscillation period has no significant effect on the mean differential sensitivities for the instantaneous concentration and its rate of change. In the case of the *swB2* ORN, however, the gain for the rate of concentration change differed significantly between the brief and the long oscillation period of 6 and 120 s.

**TABLE 1:** Summary of data used to determine differential sensitivity of the *swA* and the two *swB* ORNs at oscillating concentration changes.

Type of ORN	swA			swB1			swB2		
Duration of oscillation period, *s*	6	60	120	6	60	120	6	60	120
Range of oscillation amplitudes predominantly tested, *%*	0 - 50			0 - 50			0 - 50		
Units used for multiple regressions	10			6			7		
Number of regressions	10			6			7		
Number of points/regression	30	300	600	30	300	600	30	300	600
Coefficient of determination, *R*^2^	0.84 ± 0.16	0.64 ± 0.27	0.52 ± 0.29	0.83 ± 0.11	0.53 ± 0.21	0.42 ± 0.23	0.75 ± 0.26	0.61 ± 0.26	0.55 ± 0.29
Differential sensitivity for instant. concentration (*imps/s*)/(*%*)	0.27 ± 0.17	0.20 ± 0.15	0.15 ± 0.13	0.21 ± 0.09	0.13 ± 0.07	0.10 ± 0.06	0.15 ± 0.06	0.13 ± 0.06	0.11 ± 0.07
Differential sensitivity for rate of change (*imps/s*)/(*%/s*)	0.11 ± 0.06	0.85 ± 0.39	1.15 ± 0.90	0.27 ± 0.16	0.73 ± 0.40	1.33 ± 0.07	**0.06 ± 0.08**	0.43 ± 0.31	**0.68 ± 0.26**

*Values are means ± SD. Bold values: statistically significant (p < 0.05) differences between mean values based on ANOVA.*

### Ramp-Like Concentration Changes

**FIGURE 6:**
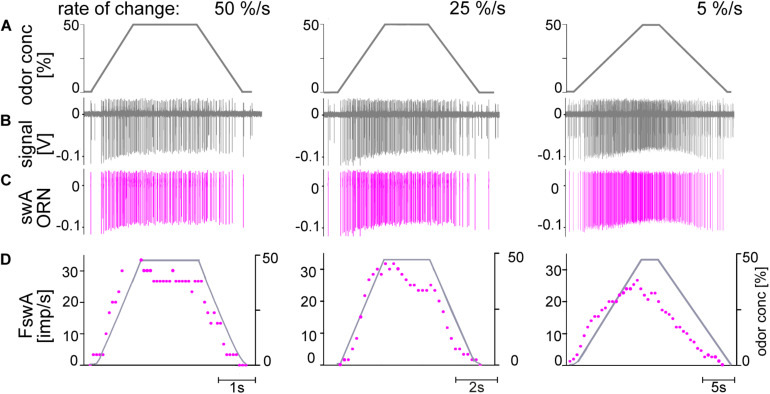
**(A–D)** Simultaneously recorded responses of two ORN located in the *swA* sensillum to ramp-like concentration changes of odor of lemon oil. Ramp rates are 50, 25, and 5%/s. **(A)** Time course of odor concentration measured by flow meter. **(B)** Recordings from the *swA* sensillum showed activities of two ORNs. While the discharge of the ORN with the smaller impulse amplitude remained unchanged (not shown in detail), the ORN with the larger amplitudes (*pink*) is activated by the slow concentration changes. **(C)** Off-line sorted action potentials of the ORNs obtained by spike detecting and template matching techniques using Spike2 software. **(D)** Time course of odor concentration and impulse frequency (bin width, 0.1 s in 50%/s, 0.2 s in 25%/s, 0.5 s in 5%/s).

**FIGURE 7:**
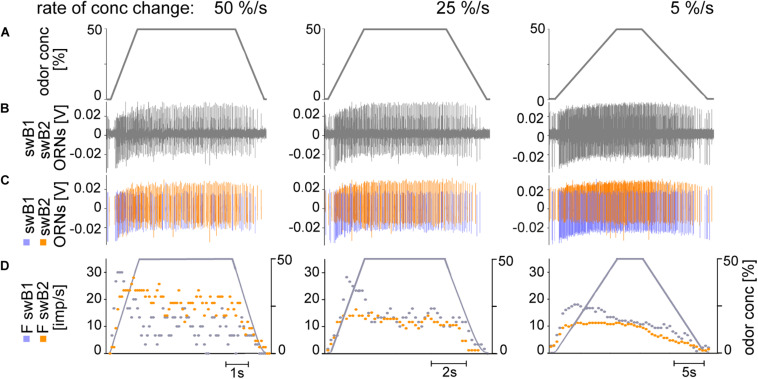
**(A–D)** Simultaneously recorded responses of two ORNs located in the *swB* sensillum to ramp-like concentration changes at rates indicated. Ramp rates are 50%/s, 25%/s, and 5%/s. **(A)** Time course of odor concentration. **(B)** Extracellular recorded activity; the action potentials from the two ORNs are different in amplitude and shape. Both ORNs are activated by slow changes in concentration. The ORN with the more negative amplitude response (*blue*) was termed *swB1* ORN and that with the more positive amplitude response (*orange*) *swB2* ORN. **(C)** Off-line sorted action potentials of the ORNs obtained by spike detecting and template matching techniques using Spike2 software. **(D)** Time course of odor concentration and impulse frequencies of both ORNs (bin width, 0.1 s in 50%/s, 0.2 s in 25%/s, 0.5 s in 5%/s).

All ORNs which produce oscillating discharge rates during oscillating concentration changes of the odor of lemon oil responded with a gradually increasing discharge rate to linear increases in odor concentration. This linear concentration increase was the upward ramp of a symmetrical trapezoid with a constant height of 50%, followed by an equally long downward ramp. The main series of experiments were conducted with upward ramp rates of 50%/s, 25%/s, and 5%/s to the trapezoid plateau (Figures 6A, 7A), each tested at least three times. Consistent with the observations made for oscillating concentration changes, only the large-amplitude ORN in the *swA* sensilla was excited by upward concentration ramps. As illustrated in Figures 6B–D, the *swA* ORN exhibited the following response characteristics: (1) briefly after the onset of the upward ramp, the ORN’s discharge increased continuously to a frequency maximum, which appeared before attaining the maximum concentration of 50%; (2) a decrease in the steepness of the ramp slope led to a decrease in the response maxima; (3) following the peak response, the discharge rate declined slowly and continuously to the initial activity, which it reached at the end of the downward concentration ramp; and (4) the magnitude of the peak response gradually decreased when the concentration rate of the ramp slope declined.

The *swB1* and *swB2* ORNs displayed the following response characteristics, as exemplified in Figures 7B–D: (1) the discharge rate increased rapidly, well-synchronized with the onset of the upward ramp; (2) during 50%/s ramps, the discharge rates of both ORNs peaked before the concentration attained the maximum value of 50%; following this phasic response, the discharge rates decreased, showing considerable variations in the decline phase; (3) during 25%/s ramps, the *swB1* ORN displayed a peak response before the concentration maximum, followed by a decrease to a highly variable tonic level for the duration of the 50% concentration plateau, then followed by a further decrease to the initial activity during the downward ramp; the *swB2* ORN discharge increased more rapidly, continued into a tonic activity with little variability until the downward ramp started, and then decreased to the initial activity, (4) during 5%/s ramps, the discharge of both ORNs increased continuously, in the *swB1* ORN more rapidly than in the *swB2* ORN, followed by a decline with remarkably low variation to the initial activity value, which was attained at the end of the downward ramp; and (5) the magnitude of the peak response of both ORNs decreased when the concentration rate of the ramp slope declined.

**FIGURE 8:**
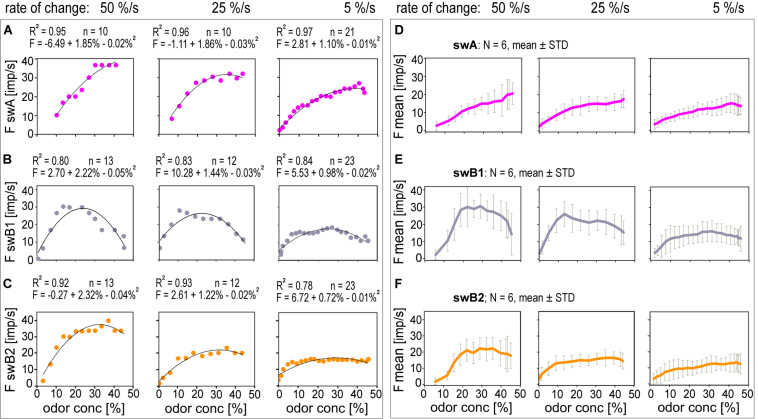
Stimulus-response functions of ORNs located in the *swA*, *swB1* and *swB2* sensilla ORNs to ramp-like concentration changes at rates indicated. Single ORNs of **(A)** *swA* sensillum, **(B)** *swB1* and **(C)** *swB2* sensillum, pooled responses of **(D)** 6 *swA* ORNs, **(E)** 6 *swB1* and **(F)** 6 *swB2* ORNs. Parabolic regressions were used to approximate the stimulus-response relationships. Bin widths for impulse counts were 0.1 s for 50%/s ramps, 0.2 s for 25%/s ramps and 0.5 s for 5%/s ramps.

A common feature of the *swA* and the *swB* ORNs was that the peak responses appeared before the maximum concentration is reached. This implies that the discharge rates did not provide information about the amplitude of the concentration change. The response profiles of each ORN type indicate differences with respect to the rate of discharge increase during concentration ramps. In Figure 8, the responses of the *swA* and the two *swB* ORNs to the three ramps shown in Figures 6D, 7D were plotted as functions of the instantaneous concentration. The individual functions were approximated by parabolic regressions. The faster the rate at which concentration increases, the greater both the increase in the discharge rate and the maximum frequency. Moreover, the faster the rate, the sooner the maximum frequency is reached. The interval between ramp onset and frequency maximum increased with declining ramp slope from 0.7 to 8.5 s in the *swA* ORN (Figure 8A), from 0.4 to 4.0 s in the *swB1* ORN (Figure 8B), and from 0.5 to 4.5 s in the *swB2* ORN (Figure 8C). By contrast, the maximum instantaneous concentration at which the frequency maximum occurs was independent of the concentration rate; it was between 32.8 and 39.9% in the *swA* ORN (Figure 8A), between 10.7 and 13.2% in the *swB1* ORN (Figure 8B), and between 15.8 and 20.0% in the *swB2* ORN (Figure 8C). The mean values and standard deviations of the samples are provided in Table 2. As is illustrated by both individual (Figures 8A–C) and pooled response curves (Figures 8D–F), the parabolas for the *swA* and *swB2* ORNs become continuously flatter with decreasing rate of change. In the *swB1* ORN, shown in Figure 8B, the parabolas change direction and open downward. As the functions become flatter, the scatter of responses around the functions decreases. Therefore, the faster the rate of change, the larger is the variability of the response. We used the differential sensitivity and the scatter of individual responses to determine the resolving power.

**TABLE 2:** Summary of data used to determine differential sensitivity and resolving power of the *swA* and the two *swB* ORNs at ramp-like concentration increase.

Type of ORN	swA			swB1			swB2		
Rate of concentration ramp, %/s	50	25	5	50	25	5	50	25	5
Range of ramp amplitudes predominantly tested, %	0 – 50			0 – 50			0 – 50		
Units used for parabolic regressions	10			6			7		
Number of parabolic regressions	10			6			7		
Number of points/regression	11–13	10–12	21–23	11–13	10–12	21–23	11–13	10–12	21–23
Differential sensitivity for instant. conc, (*imps/s*)/(*%*)	**0.79 ± 0.41**	**0.57 ± 0.38**	**0.27 ± 0.16**	1.12 ± 0.33	0.76 ± 0.28	0.40 ± 0.15	0.57 ± 0.36	0.29 ± 0.16	0.21 ± 0.08
Deviation of responses, *imp/s*	0.002 ± 0.05	0.001 ± 0.25	0.001 ± 0.03	0.05 ± 0.17	0.004 ± 0.03	0.05 ± 0.17	0.01 ± 0.01	0.001 ± 0.04	0.01 ± 0.02
Coefficient of determination, *R*^2^	0.90 ± 0.07	0.92 ± 0.04	0.87 ± 0.14	0.82 ± 0.08	0.72 ± 0.21	0.82 ± 0.11	0.81 ± 0.12	0.84 ± 0.13	0.80 ± 0.20
*F max* during concentration ramp, *imp/s*	33.42 ± 19.0	31.25 ± 17.5	23.80 ± 13.5	32.51 ± 8.21	27.28 ± 7.88	15.83 ± 5.36	21.46 ± 7.85	15.30 ± 3.17	14.17 ± 3.82
Instantaneous concentration at which *F max* occurred, *%*	32.79 ± 8.85	33.42 ± 9.64	37.78 ± 6.24	22.05 ± 8.28	15.07 ± 4.32	20.67 ± 8.85	31.02 ± 12.7	36.35 ± 9.86	38.07 ± 6.27
Time passed since ramp onset as *F max* occurred, *s*	0.73 ± 0.15	1.42 ± 0.36	7.60 ± 1.39	0.49 ± 0.10	0.83 ± 0.29	4.61 ± 1.56	0.63 ± 0.26	1.51 ± 7.60	7.60 ± 1.65
Resolving power, *%*	5.38 ± 1.47	7.06 ± 1.88	8.96 ± 3.92	8.20 ± 2.49	6.64 ± 2.27	7.42 ± 3.09	11.97 ± 7.62	9.90 ± 2.39	10.99 ± 6.91
Resolving power averaged for each ORN, *%*		7.13 ± 2.42			7.42 ± 2.61			10.95 ± 5.64	
Number of concentration steps that an ORN is able to discriminate in the 0 – 50% range		7			7			5	
Sample interval needed to discriminate the steps, *s*	0.14	0.28	1.42	0.14	0.28	1.42	0.2	0.4	2
									

*Values are means ± SD. Bold: statistically significant (p < 0.05) differences between mean values as determined by ANOVA.*

The resolving power of an ORN estimates the difference that must separate two instantaneous concentrations in order that one of them is correctly identified with a high probability (e.g., 90%) as being higher than the other. Identification is based on a single response to each concentration. The demand placed on the estimate is that the higher impulse frequency be assigned to the higher concentration. The method of determining resolving power described by Tichy et al. (2016) is employed here. The basic data are shown in Table 2. Although in each ORN type the mean values of resolving power are clearly different between the concentration rates, they are statistically not significant. Therefore, the values of resolving power were averaged over the three concentration rates and entered in Table 2. In the *swA* ORNs, the resolving power was 7% for ramps of 5%/s, 25%/s, and 50%/s; in the *swB2* ORNs the value was also 7% for the three ramps but in the *swB2* ORNs it was only 10%. The resolving power can also be defined by the maximal number of discrete concentration steps that the impulse frequency can distinguish within the concentration range between 0 and 50%. The *swA* and the *swB1* ORN can distinguish 7 steps of instantaneous concentration, and the *swB2* ORN only five steps (Table 2). The calculations revealed that the performance of the ORNs of basiconic sensilla is invariant to the concentration rate.

## Discussion

Similar to the ON and OFF ORNs located in the *swC* trichoid sensilla (Burgstaller and Tichy, 2012; Tichy et al., 2016), the ORNs in the *swA* and *swB* basiconic sensilla display a double dependence on the instantaneous odor concentration and its rate of concentration change. In contrast to the *swC* ORNs, the double dependence of the *swA* and *swB* ORNs on the two parameters of the odor stimulus was invariant to either the duration of the oscillation period or the steepness of the concentration ramp. The investigation of the performance of the *swA* and *swB* ORNs extends previous studies on the cockroach’s basiconic sensilla. We will present some background on the specific response characteristics of the ON and OFF ORNs of the trichoid sensilla and on the classification of the ORNs of the basiconic sensilla. First we describe the information on the direction and distance to an odor source that is contained in a near-ground dispersing food odor plume.

### Information Content of Near-Ground Odor Plume

Cockroaches foraging in compost areas and garbage sites, under organic mulch or leaves, can encounter all kinds of discarded food, fruits, vegetables, and decaying plants. During their foraging trips, odors fluctuate over a wide range of concentrations and odor concentration changes at various rates while they approach or leave their target food. Flying and walking plume-tracking animals deal with very different opportunities and challenges because the temporal fluctuations in an odor plume differ considerably in open air or at the ground level or underwater (Fackrell and Robins, 1982; Murlis et al., 1992; Koehl, 2006). Marine crustaceans take advantage of the direction and distance information from the spatial and temporal features of the plume (Moore and Atema, 1991; Mafra-Neto and Cardé, 1994; Vickers and Baker, 1994; Keller and Weissburg, 2004).

Odor plumes are dispersed by turbulent wind or water currents and, by expanding and mixing with the transport medium, break into pulses or patches of varying concentration separated by periods of low or zero concentration (Murlis and Jones, 1981; Atema, 1985, 1995; Zimmer-Faust et al., 1988, 1995; Moore and Atema, 1991). As the plume spreads downwind, it becomes increasingly diluted due to molecular diffusion, turbulence and topographic structures (Reidenbach and Koehl, 2011). While plume width and height increase with distance from the source, the pulse concentration and its intermittency (the proportion of time that odor is present) decreases (Murlis, 1997; Webster and Weissburg, 2001). The temporal and spatial scales of the pulses differ considerably in plumes flowing freely versus near the ground. Particular well understood is the role of the rate of concentration change in plume tracking by benthic crustaceans. On the sea floor, rapid changes in odor concentration are slowed down the nearer the odor source is to the ground, and they are damped even more when the ground surface is smooth. In near-ground plumes, the onset slope and the correlated amplitude of the odor pulses create the strongest spatial gradient pointing to the source. ORNs specialized in detecting pulse slopes and pulse concentrations were considered to be best suited for mediating direction and distance information (Gomez and Atema, 1996; Zettler and Atema, 1999). Evidence for the existence of “pulse slope detectors” has been provided by electrophysiological recordings from chemoreceptors of the lateral antennules of the American lobster (Zettler and Atema, 1999) and from the ON and OFF ORNs in a specific type of trichoid sensillum on the antennae of the American cockroach (Figure 1; Tichy and Hellwig, 2018).

### ON and OFF ORNs Located in the swC Trichoid Sensilla

The ON and OFF ORNs are activated by the same change in the concentration of the odor of lemon oil, but in opposite directions. The effective transfer of concentration increments and decrements by a dual system of ORNs profits from a single receptive field by combining them in the same trichoid sensilla. A critical function of the two ORNs is signaling plume boundaries. At the same time, their gradually and continuously changing discharge rates during slowly fluctuating concentration changes may also enable spatial and temporal sampling during odor plume tracking.

The ON and OFF ORNs display a double dependence on the instantaneous concentration and the rate at which concentration changes. Furthermore, they adjust the gain for the rate of concentration change at the expense of the gain for the instantaneous concentration. When odor concentration oscillates rapidly with brief periods, the two types of ORNs improve their response gain for odor concentration but at the same time reduce their response gain for the rate of concentration change. Conversely, when odor concentration oscillates slowly with long periods the gain for the rate of change is increased at the expense of the gain for concentration. The decrease in gain at brief oscillation periods protects against saturation, and the increase in gain at long periods improves sensitivity for slow concentration changes. In the latter case, information about the instantaneous concentration is discarded. The cue would simply be that the impulse frequency begins to change without specifying precisely the concentration level at which the change occurs. Thus, a high gain for low rates of concentration change does not correspond with a high performance for discriminating instantaneous concentration values (Tichy and Hellwig, 2018). This is an example where adjusting the sensory gain for environmental, context-dependent cues allows the cockroach to match its orientation behavior to needs imposed by slow concentration fluctuations in a plume dispersing close to the ground.

For predicting the ORN’s ability to discriminate concentration increments and their role in odor source localization, the knowledge of gain or differential sensitivity is insufficient. Differential sensitivity is defined by a regression, but the slope and height of a regression provide little clue about the extent of the cloud of points surrounding it. The differential sensitivity and the scatter of individual responses yield the resolving power – a measure for the ORNs usefulness in orientation. Tichy et al. (2016) used ramp-like upward and downward concentration changes at various rates to determine the resolving power of the ON and OFF ORNs for concentration increments and decrements. With increasing rate of concentration change, the differential sensitivity rises. Because the scatter of responses around the stimulus-response functions also increases, the resolving power for concentration increments and decrements is reduced. Accordingly, the slower the concentration changes, the greater the precision in discrimination concentration changes. The greater precision for slow concentration changes may indicate environmental priorities in odor processing. That high precision enables the cockroach to use information about the onset and offset slopes of odor pulses in addition to the pulse height to encode the inherent spatio-temporal structure of an odor signal.

### ORNs Located in the swA and swB Basiconic Sensilla

The prevailing model of insect olfaction assumes that odors are encoded and discriminated by distinct activity patterns across a population of ORNs, whereby the peripheral olfactory system of the American cockroach attracted attention. That species’ 5-cm-long, slender, tapering antenna are ∼4 cm apart at their tips, creating an “olfactory perception area” of ∼9 cm^2^. Each antenna is composed of 170 ring-shaped segments and bears 74,000 sensilla (Schaller, 1978; Altner et al., 1983). Schaller classified them into three groups – no pore (aporous), terminal pore (uniporous) and wall pores (multiporous), and subdivided the wall-pore sensilla into three types of single-walled (*sw*) sensilla and two types of double-walled (*dw*) sensilla. Two types of the single-walled sensilla (*swA* and *swB*) correspond morphologically to perforated basiconic sensilla (Figure 1), and the third single-walled sensillum type (*swC*) to trichoid sensilla (Watanabe et al., 2012). The number of sensilla varies both along the antenna, slightly rising toward the mid region, and between the animals. However, the relative number of sensillum types was fairly constant for different male cockroaches. Within the 74,000 olfactory sensilla on one antenna, coming to 68% of the total sensillum number, the basiconic *swA* sensilla make up 8% (∼6000), the basiconic *swB* sensilla 54% (∼40,000), and the trichoid *swC* sensilla 6% (∼4400) (Schaller, 1978; Altner et al., 1983). The response specificity of the basiconic *swA* and *swB* sensilla was determined by testing the female sex pheromone *periplanone*, fruits, meat, bread, cheese, and also numerous pure substances of different chemical classes which occur in natural odors (Sass, 1972, 1976, 1978; Schaller, 1978; Boeckh and Ernst, 1987). About half of the basiconic *swB* sensilla are described to be sensitive to food odors and the other half to the female sex pheromone *periplanone* (Schaller, 1978). Accordingly, 27% of the basiconic *swB* sensilla would respond to food odors. Those ORNs displayed responses to broadly overlapping spectra of natural odors (banana, apple, lemon, orange, bread, meat, cheese) and pure compounds (Sass, 1972, 1976, 1978; Boeckh and Ernst, 1987). Two types of ORNs, which respond best to octanol and terpene alcohols, are activated by the odor of lemon oil. Today, we know that the ON and OFF ORNs in the trichoid *swC* sensilla (6%) are also involved in coding of the lemon oil odor (Tichy and Hellwig, 2018). The occurrence of food-odor responsive ORNs located in both basiconic and trichoid sensilla is less an adaptation to increase the sensitivity to particular odor mixtures than to accurately process different aspects of the food odor.

### Slowly Oscillating Concentration Change

The double dependence of the ORNs’ responses became evident during slowly oscillating concentration changes. The oscillations in impulse frequencies were not in phase with the oscillations in odor concentration, but intermediary, between those of the odor concentration and its rate of change. Thus, the same impulse frequency of an ORN occurred at two different instantaneous concentrations within a given oscillation period, and the same instantaneous concentration could be accompanied successively by two different values of impulse frequency. Not only is the same response elicited at more than one concentration, more than one response is elicited at the same concentration – many in fact, since the instantaneous concentration and its rate of change are independent parameters. This does not imply that the ORNs are incapable of supplying the central nervous system with useful information on these parameters. The reason is the linear relationship between the impulse frequency and each of the two parameters. High impulse frequency signals high odor concentration. But at a given odor concentration, impulse frequency is higher still the faster odor concentration rises through the higher concentration. Conversely, impulse frequency is low at low odor concentration, but lower still the faster odor concentration falls through the lower concentration. Thus the response of individual ORNs to odor concentration is reinforced by the rate at which odor concentration changes. However, the enhancing effect of the rate of change on the response to the instantaneous concentration is invariant to the duration of the oscillation period. Variations in the steepness of the concentration slope or the range of concentration rates does not affect the differential sensitivities for the instantaneous concentration and its rate of change. Thus, characteristic properties of the ON and OFF ORNs such as gain control and the resulting trade-off between the sensitivity for the instantaneous concentration and its rate of change are not developed in the ORNs of the basiconic sensilla (Burgstaller and Tichy, 2012; Tichy et al., 2016). Unlike the ORNs of the trichoid sensilla, the sensitivity of the ORNs of the basiconic sensilla for the instantaneous concentration is neither improved when the duration of the oscillation period is reduced nor gets worse when it is extended. This favors a role in detecting odor identity.

### Ramp-Like Concentration Change

Increasing the concentration rate by steepening the linear concentration gradient caused a progressive rise of the discharge rate of the *swA* and *swB* ORNs. The maximum response rose with the concentration rate but the peak discharge occurred before the ramp attained the concentration maximum. The interval between the maximum response and the end concentration became shorter with rising ramp rate. The occurrence of the peak response prior to the concentration maximum can be explained by adaptation to the constant rate at which concentration increased. During oscillating concentration changes, no such adaptation was observed because the rate of change varies continuously over time.

Parabolic regressions were used to describe the relationship between the steadily increasing discharge rates and the linear concentration increase. Their slopes indicate the differential sensitivity or the gain for the rate of concentration change. With decreasing concentration rate the ORN’s impulse frequency tended to decrease and the parabolas to flatten. But the flatter the slopes the lower is the differential sensitivity for the concentration rate. Less evident is the effect of the concentration rate on the scatter of individual points around the individual parabolas. As inferred by the regular discharge, the standard deviations of individual responses from the parabola decrease with decreasing concentration rate. The rate of decrease, however, did not compensate for the rate with which the parabolas flatten. Thus, the resolving power of concentration increments was not statistically different for the different rates of concentration increase (Table 2). The only other insect ORN for which data on the resolving power are available are the ON and OFF ORNs in the *swC* sensilla (Tichy et al., 2016). In that case, the regression slopes remain almost constant when the concentration rate increases but the discharge loses regularity. Accordingly, the resolving power of the ON and OFF ORNs for concentration increments improves as the concentration rate diminishes.

These findings suggest that the ORNs of the *swA* and *swB* basiconic sensilla and those of the *swC* trichoid sensilla serve different functions. One proposal is that the information stream originating at the basiconic *swA* and *swB* sensilla provides characteristics about odorant identity (“olfaction-for-identification”; Tichy and Hellwig, 2018), whereas the trichoid *swC* sensilla stream is optimized to signal the timing of an odor, the rate of increase and decrease in concentration, in addition to the instantaneous concentration value. This can be understood as a specialization for mediating temporal parameters of the fluctuating concentration when tracking an odor plume to its source (“olfaction-for-action”; Tichy and Hellwig, 2018).

### Different Sensillum Types for Coding Different Features of the Odor Signal

Olfactory receptor neurons responsive to the odor of lemon oil are located in both basiconic and trichoid sensilla. The reasons for the presence of fruit-odor ORNs in morphologically different sensillum types are not known. Yuvaraj et al. (2018) concluded that the simultaneous encoding of different features of fruit odor signaling is associated with modifications of sensillum structures and alterations in the relative abundance of sensillum types. The ability of an olfactory system to detect and discriminate odor stimuli depends not only on the specificity and diversity ORNs but also on their temporal tuning properties such as the responsiveness to the rate of odor concentration change (Atema, 1995). The differences in the ORNs’ chemical response specificity and temporal tuning mean that the same odor signal encountered in the same environment can generate different sensory information. The basiconic sensilla on the cockroach’s antenna were studied much earlier with electrophysiological techniques than the trichoid sensilla. Transient pulse-like concentration changes were used to establish the chemical specificity of the ORN responses there. Rapid concentration changes were effective stimuli because, by rapidly passing the excitation threshold the discharge rate far outweighs the neural noise. In the experiments with trichoid sensilla, the problem was not to pick up action potentials from the two ORNs inside but rather to obtain reproducible sequences of action potential. Instead of transient concentration changes, slowly fluctuating concentration changes elicited smooth, antagonistic changes their discharge rates. The dichotomy between ORNs in the basiconic and trichoid sensilla appears to separate the encoding of odor identity from the temporal characteristics of fluctuations in odor concentration.

Support for different functions of the ORNs in basiconic and trichoid sensilla come from the following observations:

(1) When odor concentration increases at different rates in a ramp-like manner from 0 to ∼ 80%, the discharge rates of the *swC* ORNs of the trichoid sensilla increased gradually up to instantaneous concentrations close to the ramp plateau (Figure 4, Tichy et al., 2016). In the *swA* and *swB* ORNs of the basiconic sensilla, in contrast, a ramp-like increase from 0 to ∼50% results in a gradual increase in the discharge rate only halfway up to the ramp plateau. Note that at a ramp rate of 5%/s, the 50% plateau is reached after 10 s, and the 80% plateau after 16 s. While information about the amplitude of the slowly increasing concentration is not contained in the responses of the *swA* and *swB* ORNs, the discharge rates of the *swC* ORNs detect and encode the moment-to-moment change in concentration up to the concentration amplitude. This favors a role of *swC* ORNs in encoding temporal features of odor concentration.
(2) Due to the combined occurrence of the *swB1* and *swB2* ORNs in the same basiconic sensillum, the ORNs have the same receptive field and perceive the same instantaneous concentration at the same rate. The two ORNs respond with different strength and time course to the lemon odor. This suggests similar, overlapping chemical specificity. The classical assumption about parallel-channel coding is that information regarding both the identity and concentration of an odor is encoded as the pattern of activity across the array of ORN. The use of independent parallel channels extends the range of odors encoded by the system and transforms odor information into a spatial representation. As the same activity can be elicited in an ORN by several different odors simply by adjusting concentration, the absolute response magnitude in an ORN cannot encode unambiguously odor identity (O’Conell and Mozell, 1969). Encoding odor identity by relative activities provides a spatial activity pattern that will be constant over a range of concentrations. The two pheromone ORNs located in trichoid sensilla on the antenna of the redbanded leaf roller have been shown to produce responses that are quite variable in absolute terms but maintain a constant relationship with one another when stimulating with the primary pheromone component and its geometric isomers (O’Conell, 1975). A simple comparison of the response ratios elicited in the two ORNs of a sensillum would unambiguously encode odor identity, at least the pheromone components. Thus, encoding odor identity by relative activities provides a spatial activity pattern that may be constant over a range of concentrations.

**FIGURE 9:**
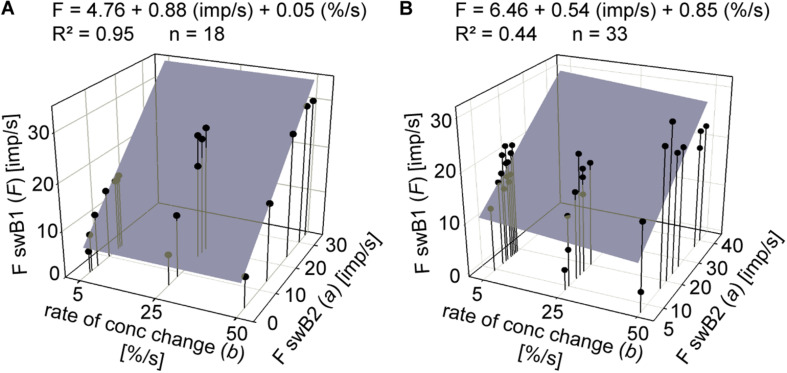
Impulse frequency of *swB1* ORN plotted as a function of impulse frequency of *swB2* ORN at the same time interval (bin width) for different rates of ramp-like concentration increase. Activity of each *swB* ORN pair was recorded simultaneously with the same extracellular electrode. **(A)** Impulse frequencies of the ORN pair shown in Figures 8B,C. **(B)** Responses of another pair of *swB* ORNs. Multiple regressions that utilize three-dimensional planes [*F swB1 (imp/s) = y0 + a FswB2 (imp/s) + b (%/s)];* where *F* is impulse frequency and *y0* the height of the regression plane] were calculated to determine the relationship of the response ratio of pairs of *swB1* and *swB2* ORNs (*a*-slope) for different rates of concentration increase (*b*-slope). *R*^2^, coefficient of determination; *n*, number of points per plot. Bin width for calculating impulse frequency specifies instantaneous concentration and the first derivative, the rate of change.

On a relative base, the *swB1* and *swB2* ORN responses offer concentration invariance. As illustrated in Figures 8B,C, the discharge rate of the *swB1* ORN increases more rapidly than that of the *swB2* ORN, and peak frequency occurs in the former prior to the latter. This relationship persists as the concentration rate varies. The rank order of the stimulus-response functions could serve as a criterion for coding odor identity that eliminates some of the ambiguity due to variations in both the concentration and the rate of change. Importantly, not only the rank order of excitation but also the impulse frequency (as a continuous variable) permits a concentration-invariant odor code. The response ratio of the two *swB* ORNs appears to be the candidate, as illustrated in Figure 9. To serve as a parameter that is independent of concentration and its rate for encoding the lemon odor, the ratios of responses to the continuously increasing concentration must be shown to be similar for the different ramp rates. The implication is not that the responses of one ORN depend on those of the other at each point, but rather that both depend at each point on further parameters, namely the instantaneous odor concentration and its rate of change.

In Figure 9A, the impulse frequencies of the *swB1* ORNs shown in Figure 8B is plotted as a function of impulse frequency of the *swB2* ORNs in Figure 8C. The range of instantaneous concentration used to plot the frequency ratios is limited to 0–20% because, at ramps at the higher concentration region, both the responses and ratio tended to decline. The example in Figure 9B shows the frequency ratios of another pair of ORNs during a larger range of ramp-like concentration increase between 0 to 40%. Even here is no indication that the ratio is affected by the instantaneous concentration or its rate of change. Note that the response magnitudes entering the ratios are absolute values in imp/s and not relative values of any individual response of an ORN type taken as a norm. The response ratio is formed from individual pairs of ORNs that are combined in the same sensillum, not from averaging the responses of several ORNs and than pairing the mean values. The response ratios of the *swB* ORNs apparently help avoid the ambiguity that concentration introduces into the activity patterning underlying *swB* ORNs. This supports the role of *swB* ORNs in odor identification.

(3) The rate of concentration change has different effects on the resolving power of the *swA* and the two *swB* ORNs compared with the *swC* ORNs. Whereas in the *swC* ORNs the resolving power of the instantaneous concentration increases as the concentration rate decreases, in the *swA* and the two *swB* ORNs the resolving power is invariant to the concentration rate. This helps the cockroach create a stable set of ORNs to encode odor identity when concentration changes at different rates.
(4) The olfactory information provided by the cockroach’s basiconic and trichoid sensilla utilizes two separate, parallel pathways to higher brain centers (Watanabe et al., 2012). The projection neurons (PNs) comprising each pathway originate in two different groups of glomeruli of the antennal lobe: the antero-dorsal (AD) and the postero-ventral (PV) group. The AD glomeruli receive the axons of the ORNs of the basiconic *swB* sensilla and transmit information via type 1 PNs to higher brain centers, the PV glomeruli are innervated by the ON and OFF ORNs located in the trichoid *swC* sensilla and connected to type 2 PNs. In the honeybee, PNs of the lateral antennal lobe tract produce fast responses to a broad odor spectrum, conveying information about the timing or temporal structure of an odor stimulus. PNs in the medial antennal lobe tract respond slowly to specific odorants and were considered to encode information about odor identity (Abel et al., 2001; Müller et al., 2002; Kirschner et al., 2006; Galizia and Rössler, 2010; Schmuker et al., 2011; Nawrot, 2012; Brill et al., 2013; Rössler and Brill, 2013; Carcaud et al., 2015). This segregation trades odor identification for concentration discrimination. The conclusion has been that the main function of parallel processing in insect olfactory systems is not to discriminate complex odor spectra but to independently extract and process different features of the odor signal. As stated above, the ON and OFF ORNs of the cockroach’s peripheral olfactory system reinforce temporal contrast information of fluctuating changes in concentration. Therefore, the two parallel pathways in the honeybee olfactory system, which probably encode and process “what” (quality) and “when” (temporal) information of the odor signal (Brill et al., 2013), support a similar functional interpretation of the two parallel processing systems in the cockroach which originate right at the antenna in the basiconic and trichoid sensilla.

### Constraints Imposed by the Rate of Concentration Change

Much of what we know about plume tracking cockroaches is based on pheromone plumes generated in laboratory wind tunnels (Willis and Avondet, 2005; Willis et al., 2008; Talley, 2010). Comprehensive and detailed studies involving the manipulation of the wind speed, the degree of turbulence, the dimension of the odor source and the visual surroundings show that cockroaches orient toward an odor source based on odor-modulated positive anemotaxis. Odor modulation indicates that the odor signal modifies the anemotactic behavior to maintain the cockroaches in contact with the pheromone plume. When the wind flow was stopped, however, the cockroaches continued to track the odor plume successfully to its source in the absence of wind, even though they took longer. Stopping the wind in the tunnel “*leaves a slowly expanding plume hanging in a zero wind environment*” (Willis et al., 2008), which indicates that anemotaxis was not used for plume tracking. Note that lobsters and crabs perform true chemotaxis in that purely the structure of the chemical signal guides orientation to the odor source (not mechanical or visual signals). In behavioral studies in an aquatic odor plume, lobsters use a spatial gradient in pulse size and shape to locate the odor source (Moore and Atema, 1991). The spatial distribution of onset slopes and the correlated pulse amplitudes provide the strongest spatial gradient pointing to the odor source. In such an “odor landscape,” odor peak height and onset slope of these peaks increase with decreasing distance to the odor source (Moore and Atema, 1991; Atema, 1996; Zettler and Atema, 1999).

Cockroaches have life styles and feeding ecologies quite different from those of lobsters and crabs, but as ground dwellers they may also use temporal odor pulse parameters during orientation along an olfactory search path. Studies of their walking behavior in wind tunnels have shown that the plume boundaries help cockroaches track the plume to the source. Navigation is thought to use information based on: (1) spatial comparisons of odor concentrations which are sampled between the two antennae or distant located receptive fields of the same antenna, and (2) temporal comparisons of concentrations which are sampled sequentially between two or more instants in time. A spatial sampling insect turns toward the ORNs detecting the higher concentration or continues straight ahead if both sets of ORNs signal equal concentration. Temporally sampling insects change the direction of their forward progress if the ORNs detect lower concentrations (Willis and Avondet, 2005; Willis et al., 2008; Willis, 2008; Lockey and Willis, 2015).

Temporal comparison of odor concentrations between two sequential samplings is limited by the resolving power of the ORNs. The resolving power is here defined as the maximal number of discrete steps that the impulse frequency can distinguish within a concentration range from 0 and 50%. The number is ∼7 for the *swA* and *swB1* ORNs, and ∼5 for the *swB2* ORN (Table 2). Although the resolving power of an ORN is invariant to rate of concentration change, the time interval that must separate two successive odor concentration samples before they can be differentiated depends on the concentration rate. In the *swA* and *swB1* ORNs, the interval is 0.14 s for a rate of 50%/s, 0.28 s for a rate of 25%/s and 1.42 s for a rate of 5%/s. In the *swB2* ORN, the values are slightly worse, 0.2 s for 50%/s, 0.4 s for 25%/s and 2 s for 5%/s. The directly comparison of the ORNs’ resolving power of the basiconic and trichoid sensilla revealed that the *swC* ON ORN are more precise at slow rates (Tichy et al., 2016). The number of steps that the *swC* ON ORN can discriminate at slow rates of 5%/s is ∼10 and the corresponding time interval ∼1.0 s; at fast rates of 50%/s, the values are ∼6 steps and ∼0.2 s intervals. Considered together, a cockroach taking sequential samples as it moves toward an odor source needs sample intervals between ∼0.1 and ∼2 s at concentration rates of 5%/s and between ∼0.1 and ∼0.2 s at concentration rates of 50%/s, depending on the type of ORN.

Male cockroaches tracking a female pheromone plume in a laboratory wind tunnel temporarily stop running and then change direction (Willis and Avondet, 2005). In different plume conditions, including zero wind, the stop duration varied between 0.13 and 0.16 s. Do the stops reflect watching for a detectable concentration increment? In view of this brief duration, the males probably use a spatial rather than temporal sampling strategy. Here we present the first estimation of the time interval necessary for an ORN with known resolving power to detect concentration increments of an air stream with slowly increasing odor concentration. In a recent investigation we have shown that the response gain of the ORNs of the trichoid sensilla is unaffected by changing the velocity of the odor-delivering air stream. The ORNs respond reproducibly to slow changes in the air stream concentration, even when the absolute number of molecules that encounter the sensillum per unit time is varied, or the air volume involved in the concentration change, or the rate of arrival of the odor molecules at the antenna, or the rate of the air flow (Hellwig et al., 2019). The finding that ORNs detect concentration changes independently of the air speed make them ideal “concentration rate detectors” by a temporal-plume-tracking cockroach.

One may argue that the time intervals, or bin widths, used to determine impulse frequency may affect the estimation of gain and resolving power. Impulse frequency may diminish as the segment is lengthened, and the regression approximating the relationship between impulse frequency and instantaneous concentration may flatten. Moreover, the deviations of the points from the regression could decrease. To counter this potential issues, we only used bin widths ranging from 0.1 to 0.5 s. Impulse frequencies for ramps of 50%/s were counted for 0.1 s intervals, yielding 10 measurements for the 1 s ramp. The bin width for ramps of 25%/s was 0.2 s, resulting in 10 data points for a 2 s ramp, and the bin width for 5%/s ramps was 0.5 s, providing 20 data points for a 10 s ramp. Note that at a rate of 5%/s, the odor concentration would change by only 2.5%/s during a single 0.5 s interval used for counting impulses. Since two concentration readings are needed to determine the amount of change, an error approaching this difference could easily be made. The accuracy of the difference could be enhanced by lengthening bin width, but at the price of lengthening the time during which all changes (other than that implied in the most general tendency) would be ignored. While we observed numerical differences in the resolving power of individual ORNs when the bin-widths were interchanged between the three ramps, the general tendency was the same.

One may also argue that the stimulus information signaled to higher brain centers by a single ORN is insufficient to account for the cockroach’s discriminative capacity to continue plume tracking and successfully localizing the odor source. This implies that the discrimination of concentration increments must depend on some integrating process that conveys to the brain the total stimulus information embedded in the response of populations of simultaneously active ORNs. We do not yet know how this integration is achieved. At this stage of knowledge, one way to attack this problem is to determine what stimulus information is represented in each of many simultaneously active ORN and what are the peripheral neural factors limiting the efficiency of odor coding.

## Conclusion

The double dependence of the ORNs on the instantaneous concentration and its rate of change appear as optimized for processing slow and continuously changing food odor concentration. Impulse frequency is high the higher concentration, but even higher when the concentration is slowly rising through the higher concentration. Conversely, impulse frequency is low the lower concentration, but even lower when the concentration is slowly falling through the lower concentration. Variations in the range of concentration rates provided by changing the steepness of a concentration wave or a gradual concentration increase had no effect on the double dependence. Therefore, the ORNs ability to differentiate concentration increments is invariant to changes in the range of concentration rates. Due to the double dependence, the ORNs function as detectors for relative rather than absolute concentration changes. As the absolute concentration is not usually a cue for plume tracking, it is advantageous to ignore it at an early stage of processing. This simplifies odor processing in the antennal lobe.

Unlike the ORNs of the basiconic sensilla, the ON and OFF ORNs of the trichoid sensilla adjusts the gain of response for both stimulus components according to the range of concentration rates. When the range of rates is large as a result of rapid concentration changes the gain for the instantaneous concentration increases and that for the rate of change decreases. Conversely, when the range or rates is small due to slow concentration changes, the gain for the rate of change increases and that for the instantaneous concentration decreases. Therefore, the ORNs ability to differentiate instantaneous concentration values diminishes when the concentration rate decreases by increasing the oscillation period or leveling off the concentration gradient. Because gain control is not developed in the ORNs of the basiconic sensilla, the effect of the rate of concentration change on the differential sensitivity for the instantaneous odor concentration is the same even if the range of concentration rates is varied. This suggests an adaptation of the ORNs of the basiconic sensilla for encoding the identity rather than temporal features of the odor signal.

The resolving power of the ORNs of the basiconic sensilla for concentration increments at linear rates of concentration increase is also invariant to the rate of concentration change. From the resolving power, we can estimate the period of time required for a temporal plume tracker to encounter concentration differences. Our analysis revealed that the ORNs in the basiconic sensilla need between 1.4 and 2 s in order to detect concentration increments when the concentration increases at a low rate of 5%/s. However, when the ORNs in the trichoid sensilla are used in temporal plume tracking, the sampling interval is briefer, namely 1 s. Accordingly, the ORNs in basiconic and trichoid sensilla are developed for different purposes: the ORNs in the basiconic sensilla provide information about the odor identity (“olfaction-for-identification”) and the ORNs in the trichoid sensilla signal the timing of the odor signal, the rate of increase and decrease in concentration, and the instantaneous value. The latter can be understood as a role in mediating information about temporal features of the fluctuating concentration during orientation to an odor source (“olfaction-for-action”).

## Data Availability Statement

The original contributions presented in the study are included in the article/supplementary material, further inquiries can be directed to the corresponding author/s.

## Ethics Statement

All the experiments described in the manuscript were performed with laboratory-reared insects. No special permit was required. After experiments, cockroaches were quickly killed by freezing. All institutional and national guidelines for the care and use of laboratory animals were followed.

## Author Contributions

MH, ML, and HT conceived and designed the experiments. ML, MH, and AM performed the experiments. HT, MH, and ML analyzed the data, interpreted the results, and wrote the manuscript. ML prepared the figures. HT edited and revised the manuscript.

## Conflict of Interest

The authors declare that the research was conducted in the absence of any commercial or financial relationships that could be construed as a potential conflict of interest.
